# Dietary Patterns, Body Composition, and Bone Health in New Zealand Postmenopausal Women

**DOI:** 10.3389/fnut.2020.563689

**Published:** 2020-10-22

**Authors:** Bolaji L. Ilesanmi-Oyelere, Jane Coad, Nicole C. Roy, Marlena C. Kruger

**Affiliations:** ^1^School of Health Sciences, College of Health, Massey University, Palmerston North, New Zealand; ^2^Riddet Institute, Massey University, Palmerston North, New Zealand; ^3^School of Food and Advanced Technology, College of Sciences, Massey University, Palmerston North, New Zealand; ^4^High-Value Nutrition National Science Challenge, Auckland, New Zealand; ^5^Liggins Institute, University of Auckland, Auckland, New Zealand

**Keywords:** foods, dietary pattern, bone health, body composition, postmenopausal women, New Zealand

## Abstract

Nutrition affects bone health status. However, analysis of the dietary patterns gives insights into which particular combination of foods may influence nutritional status and bone health. The aim of this study was to explore the associations between dietary patterns, bone mineral density (BMD) and T-scores, and body composition in New Zealand postmenopausal women. This cross-sectional study examined 125 postmenopausal women aged between 54 and 81 years. Body composition, BMD and T-scores were determined using dual-energy X-ray a bsorptiometry (DXA). Diet composition was assessed using a validated food frequency questionnaire (FFQ) composed of 108 food items, from which 34 food groups were created. Dietary patterns were identified by principal component analysis. The bone and body composition data including skeletal sites T-scores, waist circumference, BMI and body fat percentage were regressed onto the dietary patterns. Four dietary patterns were identified; the milk and milk-rich beverages dietary pattern, the dessert, cheese and red meat dietary pattern, the fruit-rich, biscuit and crackers dietary pattern and the oily fish, sports drink and seafood-rich dietary pattern. The milk and milk-rich beverages dietary pattern was significantly positively associated with spine T-score (*r* = 0.247, *P* = 0.008), and not whole-body BMD (*r* = 0.182, *P* = 0.051). The oily fish, sports drink and seafood-rich dietary pattern was marginally negatively associated with waist circumference (*r* = −0.157, *P* = 0.094) and body mass index (*r* = −0.163, *P* = 0.081) and significantly associated with body fat percentage (*r* = −0.247, *P* = 0.008). Binary logistic regression indicated that intake of the milk and milk-rich beverages dietary pattern reduced the occurrence of osteoporosis [adjusted odds ratio OR (95% CI): 0.589 (0.353, 0.982)]. A dietary pattern characterized by a high factor loading of milk and milk-rich beverages was positively associated with whole-body BMD and spine T-score, while the oily fish, sports drink, seafood-rich dietary pattern was inversely associated with total body fat percentage. Consumption of milk, even with coffee showed a positive association with bone health among postmenopausal women. Further longitudinal intervention studies is warranted to confirm effects of dietary patterns on skeletal body sites such as hip and femoral neck T-scores.

## Introduction

Maintaining bone health in older age is a public health issue/challenge especially for populations at risk such as postmenopausal women. Extensive research studies have reported the relationship between dietary factors and its effect on bone health (1–3). Consumption of high amount of fruits and vegetables (1), milk, dairy products and green tea (4) as well as a Mediterranean-style diet (5) has been associated with lower risk of osteoporosis. Likewise, consumption of low-fat plant-based dietary intervention has been linked to weight loss (6) while meat-based diets were linked to higher blood pressure (7).

The risk is heightened for postmenopausal women with estrogen deficiency related to both the early and late stages of osteoporosis (8). A modifiable mechanism of reducing the effect of menopause on older women is the consumption of a nutrient dense dietary pattern. Dietary patterns rich in nutrients such as calcium, phosphorus, protein and vitamin D have been documented to have a beneficial role for bone mass (9).

Specific diets play a significant role in determining nutritional status and health outcomes, it is therefore imperative to study what patterns of diet affect or contribute to an individual's body composition and bone health status. The consumption of a nutrient dense dietary pattern has been recognized to reduce the effects of menopause in older women. Few studies have investigated the relationship between dietary patterns and body composition (10, 11), no study in New Zealand has investigated the relationship between dietary patterns and BMD. Nutrient dense dietary pattern consumption has been reported to be associated with reduced effects of menopause in older women. Previously, we investigated the association between nutrient patterns of this cohort and their bone parameters using the 3-day diet diary and the nutrient data. We notably showed that a nutrient pattern high in the intake of vitamin E, α-tocopherol and omega 6 fatty acids may be detrimental for bone health in postmenopausal women (12). Nevertheless, we investigated the dietary patterns using the FFQ and food intake data to identify the type of foods that are significant for bone health.

In addition, there are limited studies that have explored the relationships between dietary patterns and nutritional status in New Zealand (13, 14). However, in order to better understand the relationship between dietary patterns and bone health across population in different geographical locations, data from different populations of postmenopausal women are important. Therefore, to support the search for an “ideal” diet, studies from various communities are warranted to enable generalization. To our knowledge no study has previously analyzed the relationship between dietary patterns and bone health in New Zealand postmenopausal women.

We consequently investigated the associations between dietary patterns, bone health parameters and body composition in New Zealand postmenopausal women. The aim of the study was to assess the relationships between dietary patterns and bone health evaluated by BMD site T-scores and nutritional status evaluated by body composition, BMI and waist to hip ratio in postmenopausal women.

## Materials and Methods

### Study Design

This study recruited 127 postmenopausal women aged between 54 and 81 years to participate in the cross-sectional clinical study; “Bugs'n'Bones.” The study took place in the Human Nutrition Research Unit located on Massey University Palmerston North campus from June to December 2017. We excluded two participants post recruitment, one was due to the consumption of a ketogenic diet and the other due to health conditions. G^*^Power software version 3.0.10 was used to calculate the sample size with a 90% power and an alpha of 5%.

Participants were recruited on Massey University campus by advertisement. The advertisement was also placed in a local newspaper (Whanganui Chronicle). In addition, we used a recruitment agency; Trial Facts (https://trialfacts.com/) for further recruitment. The inclusion criteria included healthy women with at least 5 years post menopause. Exclusion criteria were any presence of systemic disease such as diabetes and liver diseases, food intolerances affecting the gut, smokers and excessive intake of alcohol. Participants with any significant weight loss or weight gain (i.e., more than 5%) within the past year were also excluded. All participants were free living and apparently healthy.

All participants provided written informed consent prior to the commencement of datat collection. This study has been registered with the Australian New Zealand Clinical Trials Registry (ANZCTR) with the number ACTRN12617000802303 and was carried out in accordance with the recommendations of Massey University Human Ethics Committee Guidelines, Massey University Human Ethics Committee: Southern A, Application 17/17.

### Anthropometric and Body Composition Measurements of the Participants

Body weight of participants using the Detecto 437 eye-level weigh beam physician scale was measured to the nearest 0.1 kg. Standing height using a stadiometer was measured to the nearest 0.1 cm with participants wearing light clothes and no shoes on. Body mass index (BMI) was then calculated as weight in kg divided by height in meters squared. Waist circumference was measured by using a non-stretchable measuring tape to the nearest 0.1 cm.

Body composition measurements were measured and analyzed using the Hologic QDR series Discovery A, Bone densitometry system [Dual energy X-ray Absorptiometry (DXA)]. BMD was measured at the femoral neck (FN), lumbar spine (LS) (L1–L4), and total hip. The DXA machine was calibrated every morning for all the measurements and at the end of each day.

The reproducibility of the coefficient of variation (CV) for the DXA ranged between 0.34 and 0.70% for all measured sites. The reported lumbar spine BMD values were calculated as means of four measured values from L1–L4. The Apex System Software version 4.5.3 was used for analyzing the DXA scans. The osteoporosis classification was defined as a T score ≤ 2.5 and osteopenia as T score between −1.0 and −2.5 according to the WHO criteria (15) and were both named “OP.” The healthy classification was defined as a T-score −1.0 or greater and named “H.”

### Dietary Intake Assessment and Dietary Pattern Identification

Participants' diets were assessed with a validated semi-quantitative FFQ from the Nutrition Department, Massey University, New Zealand. Diet composition was assessed using a validated food frequency questionnaire (FFQ) composed of 108 food items, from which 34 food groups were created. The FFQ was used to collect information on participants' frequency of food intake and beverage intake. Portion size, food and beverage intake were entered into the Foodworks version 9 Xyris software which was used to analyze the participants' diet data and nutrient analyses of intake were calculated.

Principal component analysis (PCA) is an acceptable exploratory factor analysis for linking a set of variables into smaller dimensions, while varimax rotation enables an orthogonal (uncorrelated) factors that are interpretable. Validated FFQs are a gold standard method of collecting information on quantity and frequency of foods consumed retrospectively (16). Dietary assessment tools are therefore necessary for identifying patterns of an individual's diet in relation to any health issues associated.

PCA was used to identify four dietary patterns using 34 food groups collated from a 108-item semi-quantitative FFQ. The four dietary patterns were chosen based on the eigenvalues greater or equal to 2.0. A total 28.1% variance was explained from dietary pattern 1 (8.2%), dietary pattern 2 (7.8%), dietary pattern 3 (6.3%) and dietary pattern 4 (5.9%). A table of the food groups and items is attached (Supplementary Table 1). The analysis was performed for 125 study participants in order to reflect their dietary patterns. Varimax orthogonal rotation with Kaiser normalization was performed to reduce correlations between factors and increase interpretability of the results. Kaiser-Meyer-Olkin measure of sampling adequacy was 0.5 while Bartlett's test of sphericity was significant (<0.001).

### Data Analyses

IBM SPSS version 25 (IBM Company, Armonk, NY, USA) was used in this study for all statistical analyses. The outcome variables used were BMD of whole body and the regional skeletal sites T-score and the body composition parameters. The values of all variables for the whole body and regional sites were presented as mean ± standard error. The dietary patterns consisted of the food intakes such as milks (all types of milk), fruits, vegetables, yogurt/cream, white fish, red meat, coffee etc. The dietary patterns were obtained from the dimension reduction of 34 food items. We conducted binary logistic regression of the osteoporotic status (H or OP) against the dietary patterns adjusting for age, BMI and activity energy expenditure (AEE). The outcomes, i.e., bone health and body composition parameters, were regressed onto the dietary patterns generated using the “enter” method in linear regression. All *p*-values were reported significant at 0.05 or less.

## Results

The results from Table 1 illustrate that the postmenopausal women had an average age of 62 years. The mean BMI was 27.9 kg/m^2^ for the healthy and 24.9 kg/m^2^ for the women with osteoporosis. The Table 1 also shows the BMD and T-scores for the skeletal sites differentiating healthy from osteopenic/osteoporosis based on the spine T-score classification. Based on these criteria, 60 women were classified as healthy and 65 were classified as osteopenic/osteoporotic women.

**Table 1:** Characteristics of participants.

**Parameters**	**Healthy (60)**	**Osteopenic/osteoporotic (65)**	***P*-value**
	**Mean ± SE**	**Mean ± SE**	
Age (years)	62.32 ± 0.64	62.91 ± 0.49	0.463
Waist circumference (cm)	84.73 ± 1.41	77.24 ± 1.17	<0.001
BMI (kg/m^2^)	27.87 ± 0.55	24.88 ± 0.44	<0.001
Spine BMD	1.07 ± 0.10	0.82 ± 0.08	<0.001
Spine T-score	0.23 ± 0.91	−2.02 ± 0.09	<0.001
Hip BMD	0.92 ± 0.01	0.79 ± 0.01	<0.001
Hip T-score	−0.18 ± 0.90	−1.21 ± 0.09	<0.001
Femoral neck BMD	0.76 ± 0.09	0.66 ± 0.07	<0.001
Femoral neck T-score	−0.74 ± 0.11	−1.69 ± 0.08	<0.001
Total energy intake (kJ)	9269 ± 446	7608 ± 257	0.001
AEE (kJ/min)	4724 ± 1856	1516 ± 680	0.097

*BMI, body mass index; BMD, bone mineral density; AEE, activity energy expenditure; SE, standard error*.

Table 2 shows the dietary pattern factor loadings of the 34 food groups identified from the FFQ. Four dietary patterns have been named based on the factor scores high loading as shown in Table 3. The dietary pattern 1 consisted of a high factor loading of milk and milk-rich beverages. The dietary pattern 2 is high in desserts, cheese and red meat. Dietary pattern 3 comprised of high factor loadings of fruit, biscuits and crackers, potato and bread making it a carbohydrate-rich pattern. The dietary pattern 4 was composed of high factor loadings of oily fish (whole fish with fish bone), sport drinks and seafood.

**Table 2:** Factor loadings of dietary patterns.

**Foods**	**Dietary patterns**			
	**1**	**2**	**3**	**4**
Milks	**0.600**			
Vegetables	–**0.534**		0.286	−0.147
Processed meat	–**0.478**	0.188	−0.158	−0.110
Coffee	**0.462**	0.102	−0.148	−0.145
Malt and chocolate beverages (non-alcoholic)	**0.450**		0.211	
Soup	–**0.430**	−0.287	0.414	
White fish	–**0.416**		0.277	0.123
Seafood	–**0.394**	**0.367**		**0.313**
Confectionery	**0.304**	0.257		−0.105
Pizzas/burgers	0.295	0.161		−0.152
Spirits	0.249	0.227		
White meat	−0.233			−0.172
Dessert	0.129	**0.718**	0.194	
Cheese	−0.174	**0.697**	0.107	
Red meat	−0.106	**0.491**		−0.286
Sauces/dressings	−0.261	**0.425**		−0.252
Carbonated drinks		**0.387**		
Juice	0.116	**0.360**		0.183
Wine		**0.304**	−0.216	−0.129
Fruit	**0.302**		**0.467**	
Biscuits and crackers	−0.102		**0.467**	−0.104
Spreads			**0.444**	0.179
Crisps/nuts	−0.116	−0.109	**0.440**	
Tin/dry fruit			**0.401**	
Tea	–**0.312**	0.120	**0.400**	
Yogurts and cream	0.243	**0.359**	**0.373**	0.274
Rice/pasta		0.235	**0.343**	0.135
Bread			**0.338**	−0.200
Cereal/porridge	0.191		0.213	
Cake and Pie			−0.202	−0.188
Oily fish (Sardine and Tuna)		−0.181		**0.763**
Sport drinks	0.120	0.103		**0.742**
Potato	**0.333**	−0.128	**0.320**	–**0.413**
Beer				0.228
Variance explained (%)	8.2	7.8	6.3	5.9

*Extraction method: principal component analysis*.

*Rotation method: varimax with Kaiser normalization*.

*Rotation converged in nine iterations*.

*Bold value represents, Factor scores ≥ 0.300*.

**Table 3:** Logistic regression results of the association between osteoporotic status and dietary patterns after adjustment for age, BMI and activity energy expenditure.

**Dietary patterns**	**Odds ratio (95% C.I)**	***P-*value**
Dietary pattern 1	0.589 (0.353, 0.982)	0.042
Dietary pattern 2	0.710 (0.444, 1.136)	0.153
Dietary pattern 3	0.655 (0.411, 1.046)	0.077
Dietary pattern 4	0.858 (0.460, 1.600)	0.629

*Dietary pattern 1 = Milk and milk-rich beverages dietary pattern, Dietary pattern 2 = Dessert, cheese, and red meat dietary pattern, Dietary pattern 3 = Fruit-rich, biscuit, and crackers dietary pattern, Dietary pattern 4 = Oily fish, sports drink, and seafood-rich dietary pattern*.

In Table 3, a binary logistic regression of the association of the osteoporotic status against the dietary patterns was conducted. The result showed that dietary pattern 1 namely milk and milk-rich beverages dietary pattern significantly reduced the odds of being osteoporotic amongst the women. The odds of being osteoporotic was almost halved with dietary pattern 1 [OR (95% CI): 0.589 (0.353, 0.982)].

The milk and milk-rich beverages dietary pattern was significantly positively associated with spine T-score (*r* = 0.247, *P* = 0.008) but not with the whole-body BMD (*r* = 0.182, *P* = 0.051). However, this DP was not significant for hip and femoral neck T-score although a relatively high β-coefficient was recorded for femoral neck (*r* = 0.158, *P* = 0.099) and hip T-score (*r* = 0.148, *P* = 0.117) signifying a positive association (Table 4).

**Table 4:** Results of multiple linear regression of the dietary patterns (score values) and bone status^a^.

**Parameters**		**β-coefficient 95%**	**Confidence interval *P*-value**
**Spine T-score**			
Dietary pattern 1	0.247	0.094, 0.609	0.008
Dietary pattern 2	0.129	−0.071, 0.427	0.159
Dietary pattern 3	0.082	−0.138, 0.368	0.368
Dietary pattern 4	0.034	−0.202, 0.296	0.707
**Femoral neck T-score**			
Dietary pattern 1	0.158	−0.028, 0.316	0.099
Dietary pattern 2	0.016	−0.151, 0.178	0.869
Dietary pattern 3	0.034	–.134, 0.194	0.719
Dietary pattern 4	−0.091	−0.354, 0.123	0.339
**Hip T-score**			
Dietary pattern 1	0.148	−0.038, 0.334	0.117
Dietary pattern 2	0.007	−0.173, 0.187	0.937
Dietary pattern 3	−0.015	−0.198, 0.168	0.873
Dietary pattern 4	−0.082	−0.260, 0.100	0.382
**Whole body BMD**			
Dietary pattern 1	0.182	−0.020, 0.020	0.051
Dietary pattern 2	0.009	0.009, 0.049	0.925
Dietary pattern 3	0.091	−0.033, 0.007	0.325
Dietary pattern 4	−0.038	−0.031, 0.010	0.681

a *Crude model*.

*Dietary pattern 1 = Milk and milk-rich beverages dietary pattern, Dietary pattern 2 = Dessert, cheese, and red meat dietary pattern, Dietary pattern 3 = Fruit-rich, biscuit, and crackers dietary pattern, Dietary pattern 4 = Oily fish, sports drink and seafood-rich dietary pattern*.

Table 5 shows the multiple linear regression of the dietary patterns against the body composition parameters. Of note is the significant negative relationship between the oily fish, sports drinks, seafood-rich dietary pattern and the total fat percentage. High negative correlations were also observed between waist circumference, BMI and dietary pattern 4 (Oily fish, sports drink and seafood-rich dietary pattern). For the waist circumference (*r* = −0.157, *P* = 0.094) and BMI (*r* = −0.163, *P* = 0.081) and dietary pattern 4, a negative association was also observed but was not significant.

**Table 5:** Results of multiple linear regression of the dietary patterns (score values) and body composition^a^.

**Parameters**		**β-coefficient 95%**	**Confidence interval *P*-value**
**Waist circumference**			
Dietary pattern 1	−0.081	−3.034, 1.184	0.387
Dietary pattern 2	0.123	−0.676, 3.408	0.188
Dietary pattern 3	−0.043	−2.561, 1.586	0.642
Dietary pattern 4	−0.157	−3.780, 0.302	0.094
**BMI**			
Dietary pattern 1	0.032	−0.660, 0.937	0.731
Dietary pattern 2	0.100	−0.362, 1.235	0.281
Dietary pattern 3	−0.032	−0.938, 0.659	0.731
Dietary pattern 4	−0.163	−1.507, 0.090	0.081
**Total fat percentage**			
Dietary pattern 1	0.013	−1.142, 1.314	0.890
Dietary pattern 2	0.017	−1.116, 1.340	0.857
Dietary pattern 3	−0.034	−1.461, 0.995	0.708
Dietary pattern 4	−0.247	−2.901, −0.445	0.008

a *Crude model*.

*Dietary pattern 1 = Milk and milk-rich beverages dietary pattern, Dietary pattern 2 = Dessert, cheese and red meat dietary pattern, Dietary pattern 3 = Fruit-rich, biscuit and crackers dietary pattern, Dietary pattern 4 = Oily fish, sports drink and seafood-rich dietary pattern*.

## Discussion

The objective of the present research was to investigate the relationship between the dietary patterns generated from the FFQ provided by the women and their bone health status and body composition. The women were 5 years past menopause. The findings of this study showed that the high loading factor of milk and milk-rich beverages dietary pattern was positively correlated with spine T-score and whole-body BMD for the women. Binary logistic regression analysis also showed a reduction in the odds of being osteoporotic for this dietary pattern. The dietary pattern reports a high loading of milks, milk-rich beverages such as coffee. This may probably be associated to the established coffee culture in New Zealand (17). This milk and milk-rich beverages dietary pattern also had a moderate loading of fruit and yogurt and cream.

The results of this study also highlighted that a dietary pattern characterized by high factor loadings of oily fish (with fish bone), sports drink and seafood and white fish was negatively associated with total body fat percentage and marginally for waist circumference and BMI. The intake of oily whole fish with bones such as sardine and tuna as well as seafood intake was negatively associated with waist circumference, BMI and total body fat percentage. It could be noted that the intake of sports drink which may signify a moderate-vigorous activity, was also negatively related to these body composition parameters.

Although the results of this study cannot be directly compared with that of other studies due to the differences in the protocols such as the number of food group classifications and food records, the dietary patterns generated are similar to those reported previously. The finding of a positive association between dairy and fruit, and bone health was in accordance with other studies that have reported a positive relationship between dairy and fruit dietary pattern and bone health (2–4). The milk and milk-rich beverages dietary pattern in this study was similar to the dairy and fruit dietary pattern obtained by Shin and Joung (2013) from the Korean postmenopausal women (18). Similarly, this dietary pattern was comparable to the “healthy” dietary pattern obtained from Scottish postmenopausal women high in fruit, cheese, yogurt/cream (4). Of note is the reiteration that dairy foods are important for bone health.

Calcium is important for bone metabolism and is a crucial component of the bone matrix (19, 20). Milk and dairy are an important source of calcium while dairy in combination with fruit intake has been reported as a good source of iron, vitamin A, C, K, thiamin, riboflavin, niacin, and magnesium (21–24) linked to bone formation and bone health status mainly in post-menopausal women. The milk and milk-rich beverages dietary pattern would be of great benefit for bone health in post-menopausal women if improved simultaneously with vitamin D status (25). In many countries, milk is supplemented with vitamin D.

The findings of this study also suggest that a dietary pattern with a high loading of oily fish (with fish bone), sports drink, seafood, and white fish was negatively correlated with body composition most especially for total body fat percentage. The oily fish (with fish bone), sports drink and seafood dietary pattern which is similar to the Mediterranean diet was negatively correlated with total body fat percentage. The Mediterranean diet has been reported to be inversely associated with body composition (26, 27). However, sports drink featuring in this dietary pattern maybe as a result of the means by which the women replenish their electrolytes level during physical activity. The physically active women build lean body mass which has been previously positively linked to BMD (28).

Coffee is a marker of high energy and as such body composition should be taken into account for bone health related studies. Moderate coffee intake (≤400 mg/day of caffeine) appears to be potentially beneficial for metabolic health status (29). On the other hand, coffee can also be considered as a dairy (milk) marker which could have been part of the dietary pattern simply because milk is most often consumed with coffee.

Based on this research work and our previously published work on the relationship between nutrient pattern and bone health (12), our recommendation of an “ideal” diet for postmenopausal women would include intake of milk/dairy, whole grain, nuts and seeds, fruit and protein foods with less saturated fat. Quite unexpectedly, the dietary pattern 1 in this study was low in vegetables. The Mediterranean-style diet rich in the consumption of vegetables, fruit, wholegrain, fish, olive oil, and dairy products may be beneficial and is recommended (27).

The strengths of this study include the well-established data-driven statistical methodology and its high total variance explained by the four dietary patterns. The limitations of this study include its cross-sectional methodology and small sample size which could prevent causation should be taken into consideration when interpreting the study.

## Conclusion

The milk and milk-rich beverages dietary pattern was associated with spine T-score and was borderline for whole body BMD. A diet rich in milk/dairy may therefore decrease the risks of osteoporosis in New Zealand postmenopausal women. Furthermore, this suggests that no matter how milk/dairy is consumed; the impact of this type of dietary pattern on bone health especially in postmenopausal women might be more important than originally thought. Likewise, intake of oily fish (especially whole fish with bone such as sardine) and seafood may be beneficial for bone health in postmenopausal women. The study also revealed that an oily fish, sports drink and seafood dietary pattern was negatively associated with total body fat percentage.

Overall, the findings of this study suggest that there was a positive association between milk, milk-rich beverages and fruit and fish intake and bone health and may therefore decrease the risks of osteoporosis in New Zealand postmenopausal women. A dietary pattern consisting of oily fish, sports drink and seafood may have a positive influence for weight (fat) management in this population. However, further intervention research is warranted to confirm relationships between these dietary patterns and hip, femoral neck and lumbar spine T-scores in postmenopausal women.

## Data Availability Statement

The raw data supporting the conclusions of this article will be made available by the authors, without undue reservation.

## Ethics Statement

The studies involving human participants were reviewed and approved by Massey University Human Ethics Committee. The patients/participants provided their written informed consent to participate in this study.

## Author Contributions

BI-O carried out the experimental design, collected the data, conducted the statistical analyses, and wrote the first manuscript draft. JC was involved with the experimental design, data collection and supervision. NR was involved with the experimental design and supervision. MK initiated the experimental concept, was involved in the experimental design, data collection and sourced the research funding. All authors contributed to the article and approved the submitted version.

## Conflict of Interest

The authors declare that the research was conducted in the absence of any commercial or financial relationships that could be construed as a potential conflict of interest.

## References

[1] Tucker KL Chen H Hannan MT Cupples LA Wilson PW Felson. Bone mineral density and dietary patterns in older adults: the Framingham Osteoporosis Study. Am J Clin Nutr. (2002) 76:245–52. 10.1093/ajcn/76.1.245 12081842

[2] Okubo H Sasaki S Horiguchi H Oguma E Miyamoto K Hosoi Y. Dietary patterns associated with bone mineral density in premenopausal Japanese farmwomen. Am J Clin Nutr. (2006) 83:1185–92. 10.1093/ajcn/83.5.1185 16685064

[3] Movassagh EZ Vatanparast H. Current evidence on the association of dietary patterns and bone health: a scoping review. Adv Nutr. (2017) 8:1–16. 10.3945/an.116.013326 28096123 PMC5227978

[4] Park SJ Joo SE Min H Park JK Kim Y Kim SS. Dietary patterns and osteoporosis risk in postmenopausal Korean women. Osong Public Health Res Perspect. (2012) 3:199–205. 10.1016/j.phrp.2012.10.005 24159515 PMC3747660

[5] Kontogianni MD Melistas L Yannakoulia M Malagaris I Panagiotakos DB Yiannakouris N. Association between dietary patterns and indices of bone mass in a sample of Mediterranean women. Nutrition. (2009) 25:165–71. 10.1016/j.nut.2008.07.019 18849146

[6] Barnard ND Scialli AR Turner-McGrievy G Lanou AJ Glass J. The effects of a low-fat, plant-based dietary intervention on body weight, metabolism, and insulin sensitivity. Am J Med. (2005) 118:991–7. 10.1016/j.amjmed.2005.03.039 16164885

[7] Yamashita T Sasahara T Pomeroy SE Collier G Nestel PJ. Arterial compliance, blood pressure, plasma leptin, and plasma lipids in women are improved with weight reduction equally with a meat-based diet and a plant-based diet. Metabolism. (1998) 47:1308–14. 10.1016/S0026-0495(98)90297-9 9826205

[8] Riggs BL. The mechanisms of estrogen regulation of bone resorption. J Clin Investig. (2000) 106:1203–4. 10.1172/JCI11468 11086020 PMC381441

[9] Langsetmo L Barr SI Dasgupta K Berger C Kovacs CS Josse RG. Dietary patterns in men and women are simultaneously determinants of altered glucose metabolism and bone metabolism. Nutr Res. (2016) 36:328–36. 10.1016/j.nutres.2015.12.010 27001278

[10] Schrijvers JK McNaughton SA Beck KL Kruger R. Exploring the dietary patterns of young New Zealand women and associations with BMI and body fat. Nutrients. (2016) 8:450. 10.3390/nu8080450 27472358 PMC4997365

[11] Gunn CA Weber JL McGill ATand Kruger MC. Increased intake of selected vegetables, herbs and fruit may reduce bone turnover in post-menopausal women. Nutrients. (2015) 7:2499–517. 10.3390/nu7042499 25856221 PMC4425157

[12] Ilesanmi-Oyelere BL Brough L Coad J Roy N Kruger MC. The relationship between nutrient patterns and bone mineral density in postmenopausal women. Nutrients. (2019) 11:1262. 10.3390/nu11061262 31163708 PMC6628050

[13] Beck KL Jones B Ullah I McNaughton SA Haslett SJ Stonehouse W Associations between dietary patterns, socio-demographic factors and anthropometric measurements in adult New Zealanders: an analysis of data from the 2008/09 New Zealand Adult Nutrition Survey. Eur J Nutr. (2018) 57:1421–33 10.1007/s00394-017-1421-3 28378296

[14] Jayasinghe SN Breier BH McNaughton SA Russell AP Della Gatta PA Mason S. Dietary patterns in New Zealand women: evaluating differences in body composition and metabolic biomarkers. Nutrients. (2019) 11:1643. 10.3390/nu11071643 31323812 PMC6682986

[15] World Health Organization WHO Scientific Group on the Assessment of Osteoporosis at Primary Health Care Level. 2011. Geneva: World Health Organization (2013).

[16] Shahar D Fraser D Shai I Vardi H. Development of a food frequency questionnaire (FFQ) for an elderly population based on a population survey. J Nutr. (2003) 133:3625–9. 10.1093/jn/133.11.3625 14608085

[17] Tucker CM Coffee Culture: Local Experiences, Global Connections. New York, NY: Routledge (2017).

[18] Shin S Joung H. A dairy and fruit dietary pattern is associated with a reduced likelihood of osteoporosis in Korean postmenopausal women. Br J Nutr. (2013) 110:1926–33. 10.1017/S0007114513001219 23578480

[19] Shin S Sung J Joung H. A fruit, milk and whole grain dietary pattern is positively associated with bone mineral density in Korean healthy adults. Eur J Clin Nutr. (2015) 69:442–8. 10.1038/ejcn.2014.231 25351648

[20] Hardcastle AC Aucott L Fraser WD Reid DM. Dietary patterns, bone resorption and bone mineral density in early post-menopausal Scottish women. Eur J Clin Nutr. (2011) 65:378–85. 10.1038/ejcn.2010.264 21179049

[21] Calvo MS. Dietary phosphorus, calcium metabolism and bone. J Nutr. (1993) 123:1627–33. 10.1093/jn/123.9.1627 8360792

[22] Wright HH Kruger MC Schutte WD Wentzel-Viljoen E Kruger IM Kruger HS. Magnesium intake predicts bone turnover in postmenopausal black South African women. Nutrients. (2019) 11:2519. 10.3390/nu11102519 31635369 PMC6836205

[23] Orchard T Yildiz V Steck SE Hébert JR Ma Y Cauley JA. Dietary inflammatory index, bone mineral density, and risk of fracture in postmenopausal women: results from the women's health initiative. J Bone Miner Res. (2017) 32:1136–46. 10.1002/jbmr.3070 28019686 PMC5413384

[24] Hirata H Kitamura K Saito T Kobayashi R Iwasaki M Yoshihara A. Association between dietary intake and bone mineral density in Japanese postmenopausal women: the Yokogoshi cohort study. Tohoku J Exp Med. (2016) 239:95–101. 10.1620/tjem.239.95 27238552

[25] Kruger MC Chan YM Lau LT Lau CC Chin YS Kuhn-Sherlock B. Calcium and vitamin D fortified milk reduces bone turnover and improves bone density in postmenopausal women over 1 year. Eur J Nutr. (2018) 57:2785–94. 10.1007/s00394-017-1544-6 28975432

[26] Álvarez-Pérez J Sánchez-Villegas A Díaz-Benítez EM Ruano-Rodríguez C Corella D Martínez-González MA. Influence of a Mediterranean dietary pattern on body fat distribution: results of the PREDIMED–Canarias Intervention Randomized Trial. J Am Coll Nutr. (2016) 35:568–80. 10.1080/07315724.2015.1102102 27314172

[27] Mistretta A Marventano S Antoci M Cagnetti A Giogianni G Nolfo F. Mediterranean diet adherence and body composition among Southern Italian adolescents. Obes Res Clin Pract. (2017) 11:215–26. 10.1016/j.orcp.2016.05.007 27269367

[28] Ilesanmi-Oyelere BL Coad J Roy N Kruger MC. Lean body mass in the prediction of bone mineral density in postmenopausal women. Biores Open Access. (2018) 7:150–8. 10.1089/biores.2018.0025 30327744 PMC6188582

[29] de Mejia EG Ramirez-Mares MV. Impact of caffeine and coffee on our health. Trends Endocrinol Metabol. (2014) 25:489–92. 10.1016/j.tem.2014.07.003 25124982

