# Supplementary material for: Dietary Patterns, Body Composition, and Bone Health in New Zealand Postmenopausal Women
Source: Front Nutr. 2020 Oct 22;7:563689. doi: 10.3389/fnut.2020.563689 (PMC7642099; doi:10.3389/fnut.2020.563689)
Supplement: Supplementary file 1 [file Table_1.DOCX]

Supplementary Material

# Supplementary Table

Table 1. The 34 food groups and the food items included

| **Food group** | **Food items included in group** |
| --- | --- |
| Red meat | Beef, veal, lamb, pork, venison, ham, mutton and bacon |
| White meat | Chicken, turkey and other poultry |
| Processed meat | Tinned meat, pork and beef sausages, and offal |
| White fish | Cod, haddock, fish fingers and types of battered fish |
| Oily fish | Kippers, herring, salmon, tuna and tinned fish |
| Seafood | Shellfish and other sea food e.g. mussels and oyster or crab or prawns |
| Milks | All types of milk including dried, condensed and soya |
| Yoghurts and cream | Full fat and low fat yoghurts, all types of cream |
| Cheese | Full fat and low fat hard and soft cheeses |
| Potato | All potato products including hot chips/fries |
| Vegetables | Green, root, salad, peppers, onions, tomatoes |
| Fruit | All types of fresh fruit |
| Rice/pasta | Wholemeal and normal pasta, all types of rice |
| Cereal/porridge | Porridge and all breakfast cereals |
| Biscuit | Sweet and savoury biscuits, oatcakes |
| Cake | Sponge, fruit and plain cakes, pastries, pancakes and scones |
| Dessert | Milk, sponge, and fruit based desserts, custard, ice cream |
| Tin/dry fruit | All tinned and dried fruit |
| Confectionery | All types of confectionery including muesli and chocolate bars |
| Soup | Homemade, tinned and dehydrated packet soups |
| Crisps/nuts | Nuts, peanut butter, crisps and tortilla chips |
| Sauces/dressings | Bottled sauces including coconut sauce, mayonnaise, gravy and salad dressings |
| Spreads | Sweet spreads, honey, marmalade, vegemite and peanut butter |
| Coffee | Ground and instant, decaffeinated and caffeinated |
| Tea | All teas |
| Malt and chocolate beverages (non alcoholic) | Malt, barley and chocolate drinks |
| Juice | Fruit and vegetable juices and flavoured juices |
| Beer | Low alcohol and normal beer |
| Spirits | All spirits |
| Wine | All types of wine |
| Bread | All types of bread or rolls, butteries |
| Carbonated drinks | Diet and non-diet carbonated drinks and colas |
| Sport drinks | Sport and energy drinks |
| Pizzas/burgers | All types of pizzas, pies and burgers |
